# Capacity Building in Community Stakeholder Groups for Increasing Physical Activity: Results of a Qualitative Study in Two German Communities

**DOI:** 10.3390/ijerph17072306

**Published:** 2020-03-30

**Authors:** Julika Loss, Nicola Brew-Sam, Boris Metz, Helmut Strobl, Alexandra Sauter, Susanne Tittlbach

**Affiliations:** 1 Medical Sociology, Institute of Epidemiology and Preventive Medicine, University of Regensburg, 93059 Regensburg, Germany; nicola.brew-sam@ukr.de (N.B.-S.); borismetz85@gmail.com (B.M.); alexandra.sauter@ukr.de (A.S.); 2 Social and Health Sciences in Sport, University of Bayreuth, 95440 Bayreuth, Germany; helmut.strobl@uni-bayreuth.de (H.S.); susanne.tittlbach@uni-bayreuth.de (S.T.)

**Keywords:** physical activity, men, community capacity, capacity building, setting approach, health promotion, cooperative planning, participatory approach

## Abstract

Community capacity building is an essential approach for health promotion, combining a participatory approach with the view to community ownership. Little research focuses on practical capacity building strategies and monitoring. Our paper looks into involving stakeholders in facilitated group discussions as a specific strategy for fostering capacity building processes. These processes focused on physical activity (PA) promotion in two German communities (ACTION4men). Along the dimensions of capacity building suggested in literature (e.g., problem solving, resource mobilization, leadership), we implemented two participatory stakeholder groups (1/community). These groups were motivated to develop and implement PA interventions for men >50 years. For measuring capacity building processes, a semi-standardized monitoring instrument was used to document all group meetings. Additionally, we conducted semi-standardized interviews with group participants and drop-outs to capture their perspectives on capacity building. All documents were analyzed using thematic analysis. We successfully established stakeholder groups that planned and implemented a range of local measures meant to increase PA among older men. In one community, the process was sustainable, whereby the group continued to meet regularly over years. Capacity building was successful to a certain degree (e.g., regarding participation, problem assessment, and resource mobilization), but stalled after first meetings. Capacity building processes differed between the two communities in terms of leadership and sustainability. The developed interventions mainly addressed the access to organized sport courses, rather than tackling walkability or active transport. The theoretical capacity building approach was successful to develop and implement programs aimed at promoting PA. The actual capacity building processes depend upon the composition of stakeholder groups and inherent power relations.

## 1. Introduction

The community is a major setting for health promotion [1,2]. Community-based health promotion has the potential to be equitable and effective, as it is adapted to regional contexts and interacts with people in their natural living environment [1,3]. It requires concrete and effective community action in local settings, including tailored interventions taking into account local needs, assets, and opportunities [4]. To achieve this, community-based health promotion should actively involve community members such as stakeholders, professionals, key actors, and representatives of different population groups, e.g., by establishing participatory formats such as stakeholder groups or community health committees. Thus, interventions are more likely to be needs-based, effective, and sustainable [5]. The cooperation of different local actors and stakeholders can also facilitate structural changes through equitable engagement of diverse partners [6]. Stakeholder involvement is mentioned as a successful health promotion strategy in the Ottawa Charter [1] and can be used to build community capacities by creating knowledge and competencies for health promotion planning in the community [7,8]. Casey et al. [9], for example, showed that physical activity programs in communities were not sustainable (‘come and try’ events rather than structured programs) if key stakeholders were not involved. Health promoters may therefore need to move ‘the focus of their efforts... towards making other health workers and other organizations responsible for, and more capable of, conducting health promotion programs, maintaining those programs and initiating others’ [10], a process also known as ‘capacity building’. Over the last few decades, capacity building has become a central element in the theory and practice of health promotion [11]. The aim of capacity building is to empower communities (or stakeholder groups representing communities) to define, assess, and act on issues they consider to be of relevance, and to anchor health promotion programs sustainably within the community [10,12,13,14]. Building coalitions and networks, training both professionals and lay persons, as well as strengthening competence and awareness in setting members are core strategies of capacity building [15].

While literature states that community capacity building is an essential approach for health promotion (particularly for reaching disadvantaged population groups [16]), there is still little research of high quality that focuses on practical capacity building core strategies [15]. Therefore, our paper examines group involvement of stakeholders (cooperative planning) [17] as a specific strategy for fostering capacity for physical activity promotion in older adults in two German communities. Along the dimensions of community capacity building suggested in literature, we report results from stakeholder group meetings, their participatory planning and decision making, as well as activities intending to implement physical activity interventions for men over 50 years of age (50+) in the two communities. The study (“ACTION4men”) is part of a large-scale German transdisciplinary study network “Capital4health”, which engages stakeholders in different settings to foster an active lifestyle [18,19].

In Germany, four out of five adults do not achieve the WHO recommendation of at least 2.5 h/week of moderate-intensity physical activity [20]. Yet, the beneficial health effects of regular physical activity are well documented [21,22,23,24]. Physical inactivity is an important risk factor for many chronic diseases (e.g., diabetes, hypertension, osteoporosis, or various types of cancer), and also relates to increased healthcare costs [25], whereas regular physical activity has potential to reduce disease progression [26]. Levels of physical activity decrease with age [27,28], making older adults more prone to chronic diseases. The literature shows that older and elderly people can benefit considerably from regular exercise–from low intensity walking to more vigorous sports—as it decreases their risks of developing major cardiovascular and metabolic diseases, osteoporosis, falls, and cognitive impairments. Even for those people who were sedentary through middle age, health can be significantly improved by becoming more physically active in older age [29,30]. An increase in moderate physical activities induced through a horticulture program in older adults (60–77 years) was found to reduce levels of biological markers associated with chronic diseases (e.g., pro-inflammatory cytokines) [31]. Community-based capacity building interventions have been shown to be particularly promising for the promotion of physical activity in older adults, yet further studies are still required to get a clearer picture on results of community capacity building strategies aiming at older target groups [15].

Additionally, literature points out that physical activity programs need to be gender-sensitized in order to be successful [32]. Physical activity differs by gender, with men reporting higher physical activity than women in various age groups [27,28,33]. Yet, research data show that an alarming proportion of older men do not reach recommendations for physical activity [34,35] and that existing health-enhancing physical activity programs often fail to reach and attract older men. Physical activity barriers perceived by older men include fear of injury or existing physical constraints, lack of meaningful physical activity possibilities (e.g., regular programs), environmental constraints (e.g., lacking facilities), or lack of time [36,37]. Literature states that men respond well to community-based physical activity programs when gender-specific promotional and delivery strategies are used [38,39,40].

### Study Aims

(1) To establish stakeholder groups in two communities that are willing to meet regularly to plan exercise interventions for older men.
(2) To monitor and examine the capacity building processes that take place in these stakeholder groups, in order to understand whether participatory approaches on the community level are effective in developing and implementing local physical activity interventions.

## 2. Materials and Methods

**Figure 1:**
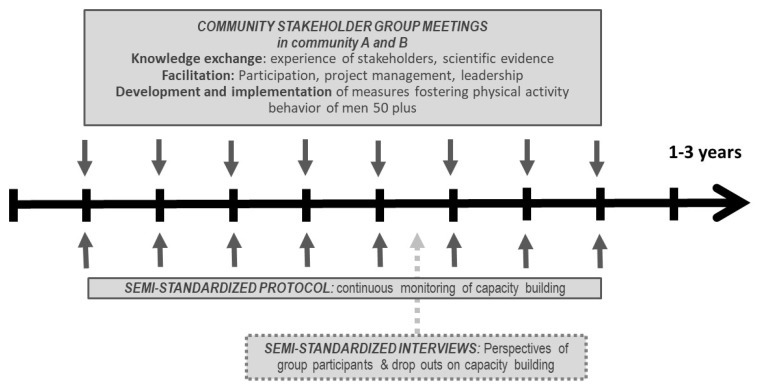
Capacity building and evaluation in ACTION4men.

Figure 1 displays the research procedure and gives an overview over the research methods applied. The exact procedure is addressed in a study protocol [8]. The participants of the study are community members who volunteered to join a participatory stakeholder group, which would plan and implement interventions fostering exercise among older men. Those interventions are described in the manuscript, but they were not systematically evaluated in terms of acceptance, reach and/or effectiveness; the focus of the study was the capacity building process within the stakeholder groups.

### 2.1. Community Stakeholder Groups

Two rural communities (20,000 and 10,000 inhabitants, respectively) in Bavaria, Germany were chosen as study areas, based on several selection criteria. These selection criteria included the community size (5000–25,000 inhabitants), existing infrastructure (sport clubs, clubs addressing predominantly men, e.g., male choir), formal support by the mayor and community council, and health data showing an increased mortality rate for men in this region [41]. In both communities, we addressed stakeholders from a variety of sectors (e.g., municipality council, sport and non-sport clubs, health sector, etc.) by personal written invitation letters as well as newspaper articles pointing out a first information event, to which also the broad public was invited. People attending the information event were asked to join a community stakeholder group, which was meant to plan and implement physical activity measures for men 50+ in a participatory planning process (‘cooperative planning’) [42]. As this was a community-based participatory process that invited everybody interested in this issue, there were no inclusion or exclusion criteria. Any local stakeholder or citizen that wished to participate in the group meetings could become a group member. The researchers involved in this project were specialized in health promotion and supported the stakeholder group activities throughout the whole project duration. They facilitated regular stakeholder group meetings (8–30 participants), fostered the development of relationships with other stakeholders, offered knowledge input, and engaged in a stepwise transfer of responsibilities regarding the development and implementation of physical activity measures to the stakeholder group. The health promotion researchers also fed back results from a mixed-methods needs assessment in men 50+ [8] into the stakeholder groups.

### 2.2. Assessment of Capacity Building

#### 2.2.1. Semi-Standardized Monitoring Protocol

For measuring capacity building among the stakeholder groups, we used a semi-standardized monitoring instrument validated by Sauter et al. [43]. It is based on what Liberato et al. [13] call the ’Goodman/Labonte/Laverack/Fawcett model’ of capacity building [44,45]. Representing this model, Laverack [44,46,47] summarized nine dimensions of community capacity building based on previous conceptualizations (e.g., dimensions by Hawe et al. [10,48], Goodman et al. [49]). Similar domains have been used and proven useful in studies evaluating capacity building outcomes and health outcomes [50,51]. Table 1 describes the nine capacity dimensions in detail. The instrument used is available for download (https://www.uni-regensburg.de/medizin/epidemiologie-praeventivmedizin/medizinische-soziologie/index.html).

For each meeting, detailed descriptions of the stakeholder group’s developments according to the nine domains were made. At least two health promotion researchers participated in each stakeholder group meeting; both of them filled out the monitoring instrument independently and then discussed their perspectives. The contents of the versions of both researchers were merged to a final version.

#### 2.2.2. Semi-Standardized Interviews

Additionally, we conducted semi-standardized face-to-face interviews with members and drop-outs of the stakeholder groups to receive additional information about the process of community capacity building. The interview guide was equally based on the nine capacity domains of capacity building shown in Table 1. A total of 13 interviews were conducted (see Table 2 for functions/roles of interview partners). In both communities, the interviews were carried out after one year of project duration/stakeholder group meetings (Community A: After termination of the stakeholder group meetings; Community B: After twelve months of the stakeholder group meetings). Overall, data was retrieved from 23 monitoring tools and from 13 semi-standardized interviews (see Table 2).

### 2.3. Data Analysis

For data analysis we followed the standards for qualitative research by Mays and Pope [52], and used thematic analysis as described by Braun and Clarke [53]. The aim was to use an existing theoretical foundation (capacity building concept) for qualitative data analysis rather than develop a theory from the data (as found in grounded theory) [53]. In thematic analysis, themes or patterns are searched for across a data set (here: Meeting protocols and interview data). A theme was considered a topic deriving from data content, with key themes deriving from the nine theoretical capacity domains, and sub-themes developed dynamically from the meeting and interview data. The data analysis included several steps, as described in Braun and Clarke [53]. The overall process was dynamic and iterative. The interview transcripts were read and coded independently by two health promotion researchers. Deviant cases and contradictory data were analyzed with particular attention and discussed within the research team [52]. The coding software ATLAS.ti Version 7 was used for performing axial coding in order to identify relevant text passages from interview and meeting data.

## 3. Results

The processes and outcomes of capacity building in both communities are described according to the different domains listed in Table 1. For a better understanding of the described proceedings and developments in the respective stakeholder groups, the sub-chapter ‘Organizational structure’ also contains the description of the different interventions that were planned and implemented by the stakeholder groups.

### 3.1. Organisational Structure and Implementation of Physical Activity Interventions

Stakeholder groups could be recruited successfully in both communities, and they met regularly over several months (see Table 2). The stakeholders participated in the group meetings mainly because of a certain professional function of theirs (e.g., sport club representative), but the majority happened to be male and older than 50 years, thereby also representing characteristics of the target group. During the course of the meetings, the stakeholder groups in both communities developed and implemented a number of activities aimed at increasing physical activity of older men (see Table 3). Whereas the meetings of the stakeholder group in Community A were discontinued after 11 months, Community B maintained regular meetings with the facilitating researchers over 30 months and eventually employed and funded a person (a male retired police officer who used to be a sports trainer) to manage the stakeholder group and interventions.

### 3.2. Participation

In both communities, a broad range of disciplines and representatives participated in the first meeting, but the heterogeneity dwindled over time. Around the second or third meeting, many participants had dropped out (e.g., pharmacist, gym owner, fire brigade representative). Finally, the groups had a stable composition comprising sport club representatives and members of the community administration; in Community A, also a doctor and a representative of a local company kept attending the group meetings. In both communities, the sport clubs and their topics dominated the discussions and focus of the meetings. They clearly considered the issue ’physical activity of older men’ to be their area of expertise and responsibility. This contributed to a certain efficiency in the planning and implementation processes.

*“The nice thing about [the meetings] was the clubs haven’t thought of each other as competitors, but have worked together, and actually, in the joint work, found a solution [i.e., the SportCard].”*

(IP 10, sport club representative, Community B)

The dominance of the sport clubs was also the main reason other participants decided to stop attending the stakeholder group meetings, as they felt their perspectives or needs were neglected, or because they perceived the interactions as competitive.

*“[The gym I run is] not a sport club, strictly speaking, and therefore the gulf was just too big between [me and] the clubs, which kept presenting their approaches and programs. That was difficult for me … because everyone only wanted to enhance their own interests.”*

(IP 13, Community A, owner of a private gym, dropped out after meeting no. 2)

*“For us it quickly became clear that [cooperation with other actors] would be almost impossible, for organizational …reasons.”*

(IP 05, Community A, representative of adult education center, dropped out after meeting no. 2)

*“I felt like I wasn’t noticed at all …The Workers’ Comradeship Association, we’re not a sport club.... Those sport clubs stepped forward and more or less, [they] just wanted to present their [respective] clubs to the group.”*

(IP 4, Community A, representative of Workers’ Comradeship Association, dropped out after meeting no. 1)

Some stakeholders appreciated the participatory approach, whereas others felt it would delay the process. Some would have preferred a more directive approach.

*“On your (i.e., the facilitator’s) part, that’s to say on the part of the university, there has been a willingness to deal with this topic [implementation of physical activity interventions] in a very open-minded way, you were very open-minded, and you seriously considered different propositions. I felt it was always focused on solutions.”*

(IP 10, Community B, sport club representative)

*“The [procedure of the stakeholder groups] was a bit unstructured, and I think the participants had too much of a say.”*

(IP 02, Community A, sport club representative).

*“Mr. X [participant of the stakeholder group] seems to be impatient during the meeting [with much discussion]...’We now need a structured procedure to move this group forward!’... He said he didn’t see that anything had gone ahead in this meeting.”*

(monitoring protocol, meeting no. 3, Community B)

### 3.3. Problem Assessment and Solution

The facilitating researchers could easily raise the stakeholders’ awareness that older men may have specific needs in terms of physical activity. In both communities, the stakeholder groups also came to discuss about and agree on certain barriers, e.g., that men may have difficulties in identifying courses suitable to their needs, or that a (long-term) membership in a sport club may discourage men from trying out sport courses. The stakeholder groups were adept and efficient in finding and implementing solutions for overcoming these hurdles, e.g., the SportCard or the Website. Beyond these circumscribed aspects, the stakeholders in both communities seemed reluctant to discuss or analyze other potential problems and barriers in their communities. There was no discernible interest to understand in depth different possible causes for older men’s inactivity, and tackle those, among the members of the stakeholder groups.

*“[The] sport club representatives …particularly keep an eye on their interests, i.e., promotion of their clubs to potential members. The wider perspective…, i.e., encouraging (inactive) men to exercise more often and to implement innovative approaches, is not overtly supported by the representatives of the sport clubs.”*

(monitoring protocol, meeting no. 9, Community B)

Once they had implemented the first interventions, the stakeholders used their efforts to maintain and continue these interventions rather than shifting their focus to problems still unaddressed. The facilitating health promotion researchers kept encouraging the stakeholder groups to consider novel interventions, and regularly pointed out other potentially relevant needs, e.g., by presenting survey and interview results from the needs assessment among local men. These inputs were not taken up or pursued further in a serious way by the stakeholder groups. Consequently, some smaller interventions were planned in additional bilateral meetings with interested participants outside the group meetings (the Outdoor Activity Meetings, and a survey in the local company), and then presented back to the stakeholder group.

*“The participants of the stakeholder group don’t discern a clear benefit of the Outdoor Activity Meetings for themselves, and therefore are not too interested in this intervention. The mayor …makes clear that [the municipality] will financially support the Outdoor Activity Meeting, but the organization had to be done by the researchers and those group members interested in this intervention.”*

(monitoring protocol, meeting no. 9, Community B)

### 3.4. Critical Awareness and Reflexivity

In Community B, the stakeholder group repeatedly reflected and discussed how the SportCard and novel sport courses tailored to men were accepted and used by men 50+. These assessments were initiated by the facilitating researchers, and in the end were often restricted to an individual cost-utility analysis for the respective sport club. Other than that, the stakeholder groups in both communities did not tend to question the usefulness or effectiveness of the interventions they had planned; they appeared to be very satisfied with the achievements. Some single participants were asking to consider that the interventions may fail to reach men not interested in sport club courses, but these suggestions were overruled or dismissed by the majority (e.g., meeting protocol no. 12, Community B), for example, because they were seen as issues of the facilitating health promoters.

### 3.5. Resource Mobilization

In both communities, the stakeholder groups could easily mobilize resources that were needed for the implementation of the interventions. This included fundraising for the production and printing of the leaflets, identifying instructors for the Outdoor Activity Meetings, and securing venues for the group meetings and kick-off events. In Community B, where the mayor was a regular participant of the stakeholder groups, the municipality was willing to finance many of the interventions, e.g., subsidizing the SportCard or paying the fees for the trainer of the Outdoor Activity Meetings.

### 3.6. Links to Others

Beyond short-term contacts meant to mobilize certain resources, the stakeholder groups built coalitions with other sport clubs and the press. For example, further sport club representatives were recruited to contribute to the SportCard intervention (Community B). In addition, the senior citizens’ representative established a contact to an umbrella sport organization in order to discuss whether the developed interventions could be transferred to other communities. Beyond that, the stakeholder groups were reluctant to seek out coalitions or networks with other institutions. The stakeholder groups would not know whom to address in existing local non-sport clubs with many male members, and were doubtful that a potential link to those clubs would be relevant.

*“The [facilitating] research team proposes to contact other organizations in which many men usually come together, but this is not considered necessary by the group members present at the meeting. The stakeholder group cannot name any adequate contact persons at those organizations either.”*

(monitoring protocol, meeting no. 10, Community B)

### 3.7. Leadership

With regard to leadership, the stakeholder groups developed in different ways. In Community B, the mayor took a leading role within the stakeholder group from the beginning. He set the agenda of the meetings and managed these in an efficient way.

*“With his input, the mayor has contributed to putting [our interventions] on a successful track, the SportCard for example.”*

(IP 10, Community B, sport club representative)

This leadership, however, happened at the expense of a stronger participation of the other group members and their ideas. When a former sport trainer was appointed a part-time job in the council to organize and run the activities of the stakeholder group, the leadership role was transferred from the mayor to this new person. In Community A, the members were rather passive in most meetings, and relyed on the health promotion researchers to delegate tasks and manage the interventions that they had come up with. No considerable leadership could be developed over time.

*“At present, it seems unrealistic to form a stakeholder group that operates independently of us researchers. The [stakeholder] group continues to expect us, i.e., the facilitating researchers, to cover and manage all topics, be they organizational or related to the agenda of the meetings.”*

(monitoring protocol, meeting no. 6, Community A)

### 3.8. Relationship with Outside Agents/Program Management

The role of the facilitating health promotion researchers (the outside agents) shifted in both communities over the time. In the beginning, i.e., the first (three) meetings, the researchers were in control of the meetings (they set the agenda, presented background information about men’s specific needs, and suggested ideas for interventions), and discussed the given input with the stakeholder groups. In the subsequent meetings, both stakeholder groups came up with their own suggestions for interventions rather quickly, and decisions were made jointly with the researchers. After the implementation of the first intervention (leaflets and website), the stakeholder group in Community A returned to a more passive state, mostly waiting for the researchers to present ideas that they could discuss. In Community B, the stakeholders developed some further strategies (e.g., the Sports Insignia) and decided on the necessary steps rather independently with only slight support of the researchers.

As to project management, the research team was seen as responsible for most of the (organizational) tasks. Only few operational steps were taken over by participants of the stakeholder groups.

*“For the first time, a subgroup is founded, consisting of members of the stakeholder group, dedicated to a specific task: the organization of the Sports Insignia activity. Participant Z addressing the [facilitating] researchers: ‘We’ll do it, you don’t have to worry about it.’”*

(monitoring protocol, meeting 9, Community B)

*“The mayor communicates that he would financially support the planned Outdoor Activity Meetings. The organizational jobs, in turn, would have to be get done by the facilitating researchers: ‘It’s your project’”*

(monitoring tool, meeting 9, Community B)

In Community B, it was eventually decided to employ a local person to take over the tasks of the researchers to sustain the project work in the community.

Similarities and differences in the capacity building processes between the two communities are highlighted in Table 4. 

## 4. Discussion

### 4.1. Principle Findings

In two communities, we could successfully establish participatory stakeholder groups that planned and implemented local measures for increasing physical activity among older men. Over the course of time, the groups were mainly dominated by representatives of sport clubs and their interests; other stakeholders were often less active in the meetings, or stopped attending the group meetings altogether. In one community, the stakeholder group discontinued their meetings once the first interventions were implemented, as they felt they had satisfyingly fulfilled their task. In the other community, the process became sustainable as the stakeholder group continued to meet regularly and the council employed a person for the long-term management of the program. Capacity building processes were successful to a certain degree (e.g., in terms of participation, problem assessment, and resource mobilization), but stalled after the first meetings. The stakeholder groups already disposed of a relatively high level of certain capacity domains from the beginning. For example, sport club representatives were familiar with implementation and marketing of sport courses and activities, and the mayor had leadership characteristics. In Community B, this resulted in the formation of a group that worked independently (without guidance by the facilitating researchers) and kept implementing an array of sports activities. The downside of this relatively high capacity level was that the groups (and their members) could hardly be convinced of a need to develop their capacities further, e.g., sport club representatives were experts in sport courses, but many of them would not see it as their task to learn more about how environmental changes in the community could increase physical activity, or how to reach men who are deterred by formally organized courses. It was difficult for the facilitating researchers (and single participants) to stimulate critical reflections without being mistaken for discrediting the efforts and achievements made by the stakeholder group. Consequently, the range of interventions was mainly restricted to increasing the access to organized sport courses, rather than improving walkability, active transport or social support for physical activity in the communities.

### 4.2. Strengths and Limitations

By detailing the course of the stakeholder groups’ activities and the capacity building processes, our study makes transparent and traceable how the composition of a group and the dynamics of interactions influence group development in terms of competencies, power, and effectiveness. The meticulous monitoring and reflected documentation of the meetings, supplemented by interviews with the group members and drop-outs, yielded a comprehensive and rich data set. It is a limitation, however, that we only report on the experience in two communities. Given the flexible and emerging nature of participatory projects, the same approach will most probably yield different proceedings and results in different communities. Indeed, the capacity building processes significantly differed between the two stakeholder groups in terms of leadership and sustainability. Still, certain phenomena were alike in both studied stakeholder groups, e.g., the dominance of sport clubs and the resulting focus on interventions improving access to courses offered by those clubs, or difficulties in raising critical awareness about the groups’ own achievements. One needs to bear in mind that the findings from this study cannot simply be transferred to other countries or cultural contexts; they may reflect a specific German situation. Considering the significantly different historical, political, and cultural backgrounds of states all over the world, considerable differences in implementing public participatory processes can be expected.

This study focused on the capacity building processes among community stakeholders. It was beyond the scope of the study to analyze the effectiveness of the sports activities that were developed and implemented by the stakeholder groups. Therefore, we have not assessed the impact of the measures (e.g., SportCard, leaflets) on the exercise behavior and health of the target group of older men. A pre–post survey in Community B is currently being performed to evaluate changes in physical activity [8]. In addition, it would be interesting to measure changes in anthropometry as well as biological markers, such as brain-derived neurotrophic factor [54], in those older men who took part in the various exercise offers that were launched in the two communities.

### 4.3. Comparisons with Other Studies

To our knowledge, this is the first study using the capacity building approach to develop and implement programs to promote physical activity of older men. Other studies used community capacity building approaches mostly for other target groups, other health promotion fields, or use core strategies differing from stakeholder group meetings [16,50,55].

A Belgium study highlighted the importance of political involvement on capacity building through partnerships with local stakeholders [16]. In qualitative interviews with local actors, the health promotion researchers found that the support of politicians and policy appeared to be critical for funding and legitimacy of community sport programs. Our study confirmed the relevance of representatives from politics for group outcomes; the inclusion of leading decision-makers (e.g., mayor of the communities) was found to positively support the legitimacy of the intervention and the implemented physical activity measures, as well as guarantee additional resources.

A Canadian study compared the process of capacity building in two community sport organizations [55], and showed how a similar capacity building approach in different contexts can have differing results; one organization could successfully build capacity on a large scale and implement a new program, however, the other organization failed. The authors attributed this to differences in the already existing capacity. These results resemble our own observations, showing that in Community B, the capacity building processes were more successful and sustainable than in Community A.

Another Canadian research group investigated the process of community capacity building for chronic disease prevention [56] using interviews with health promotion practitioners. They confirmed that the success of capacity building relied largely on a number of factors, such as existing capacity, the coordinator role, or community connections. In our study, there were mixed results about the role of the existing capacity level for the capacity building process; while relatively high levels of capacities in planning and management made the group process more efficient, these also seemed to lower the participants’ readiness (or perceived need) to develop other capacities.

Moreover, trust can be a relevant factor influencing capacity outcomes, with higher trust resulting in better collaboration. Marlier et al. [16], for example, showed that trust resulted from a longer period of collaboration, personal contact, and clear coordination. Trust can also motivate participation of stakeholders in the group meetings. While we have not explicitly included ‘trust’ in our monitoring tool or interview guide, it transpired that a perceived lack of respect or lack of appreciation induced some participants to discontinue their participation, e.g., the representatives of Workers’ Comradeship Association, of a private gym and of the adult education center.

### 4.4. Implications for Policy and Practice

When intending to implement local interventions for promoting physical activity among older adults, it could be effective to establish participatory stakeholder groups in communities, according to our study. Our results also imply that sport clubs tend to dominate these groups and the interventions planned in the groups, notwithstanding our efforts to include a wider variety of stakeholders in the group and to support those group members not representing a sport club. Bearing this phenomenon in mind, health promoters may need to complement such ‘official’ stakeholder groups by additionally using other participatory approaches, e.g., bilateral cooperation with voluntary organizations and non-governmental organizations (NGOs), quality circles with health professionals, or empowerment approaches among (inactive) community members, or in sub-settings [57,58]. Thereby, they could lower the threshold for those stakeholders and citizens who do not have clear vested interested in sports, but may be more open-minded about, and bring up, further ideas regarding information on physical activity, walkability in the community, or ‘unconventional’ opportunities for movement.

For the facilitating health promoters, it was challenging to maintain a balanced participatory discussion in which every group member felt respected and appreciated. The reasons were manifold and inherent in the structure of the groups and community history; there were power imbalances (leading politicians vs. representatives of smaller clubs), there were quantitative imbalances (representatives of sport clubs outnumbering representatives of any other club), and there were existing resentments (e.g., between public and private sector, between sport clubs and adult education center). Studies on inter-sectoral partnerships in the Healthy Cities Project and on community capacity building have also noticed that “traditional divisions and power relationships… [may be] maintained”, and significant tensions and competitive thinking may exist within partnerships [15,59]. Facilitators and coordinators of capacity building processes in community stakeholder groups should acknowledge the need to: understand the roles and requirements of all participants; invest much energy in building a stable coalition between participating stakeholders; and accept the limits within which common visions and shared ideas can be developed [59,60]. The facilitators may also need to be prepared that “building relationships, developing trust and strategizing around a range of (…) personal (…) and organizational challenges” may take up considerable time [61]. Following a more purposive recruitment strategy, rather than inviting anyone who is interested in the topic, could counteract quantitative imbalances [15]. For example, one could intentionally ask only one representative of each sector to participate in the stakeholder group (sport club, private gym, non-sport club, council). This procedure may create a more effective stakeholder group, though at the cost of broader community participation.

The two groups consisted of stakeholders who were relatively experienced in (organized) sports, and long-dwelling community members. We found that in these groups, some capacity dimensions could be built more easily than others. On the one hand, the groups succeeded in establishing a regular meeting schedule (“organizational structure”), could develop effective plans meant to motivate older men to be physically active (“problem assessment and solution”), and were adept at raising funds or recruiting personnel for different interventions (“resource mobilization”). On the other hand, the groups were reluctant to take over responsibility for many operational tasks (“program management”), to reflect critically upon their own achievements (“critical awareness”), and to seek out novel cooperations (“links with others”). This observation may partly be attributed to the fact that the stakeholder groups—although they were facilitated in a flexible, participatory way inviting bottom-up initiatives—were set up as a ‘top down’ approach initially. The basic agenda (i.e., planning community interventions that make it easier for older men to be physically active), was determined by the facilitating health promoters, in line with the focus of the funded research project. This may have, at least in Community A, supported the notion among stakeholders that they were ‘fulfilling a task’ given by the external health promoters, rather than developing their own vision or agenda. A few stakeholder comments, such as ‘this is your project’ or ‘this is your problem’, hint at a certain mismatch between the interests of the stakeholder groups and the facilitating researchers. Laverack [62] acknowledges that in health promotion and disease prevention, there is often a duality of a professionally led programming, based on epidemiological evidence and best practice, and a ‘bottom-up’ approach of the community, which address the concerns of the beneficiaries (or, in our case, the stakeholders). He suggests a framework called ‘parallel-tracking’ in order to accommodate bottom-up objectives within top-down priorities of programming. In our case, a parallel-tracking procedure was followed in Community B where the stakeholder group was focused on developing and sustaining interventions related to sport clubs, and one participant of the stakeholder group (together with the facilitating researchers) pursued the idea of an independent low-threshold outdoor meeting on their own. The facilitating researchers still supported the stakeholder group’s preferences, while also keeping the group informed about the progress of the Outdoor Activity Meetings, which eventually convinced the stakeholder group that this was also an intervention with significant merit. This parallel-tracking approach can help maintain certain evidence-based purposes of a program, while at the same time supporting stakeholders’ needs and building their capacity [62].

We have listed the activities planned and implemented by the participatory stakeholder groups as outcomes of the capacity building process. In addition, we also learned (mostly by chance) about further unplanned impacts of the project. For example, other communities in the region started implementing their own SportCard interventions after they had become aware of the activities in Community B. In addition, the adult learning center in one community (whose representative dropped out of the stakeholder group) added to its portfolio novel training courses targeting older men. These unexpected impacts (also called ‘ripple effects’ of an intervention) have not been evaluated systematically. For future research on participatory community approaches directed at physical activity promotion, it may be interesting to follow up on these unanticipated effects, as they have the potential to change the context and the determinants of health more than the ‘narrowly focused outcome goals of projects’ [63].

## 5. Conclusions

Participatory stakeholder groups in communities can become key actors in implementing local interventions with the aim of promoting physical activity among older adults. There can be a tendency for representatives of sport organizations to join these groups predominantly. Therefore, coordinators may need to invest much energy in maintaining a balanced discussion among group members, and to keep other community representatives motivated to participate and have a say in capacity building processes and intervention planning. Coordinators should also strive to win the active support of a representative of the authority as part of the stakeholder group, who can champion the processes and has the power to make them more sustainable. For the evaluation of the stakeholder group’s output, it would be interesting to develop measures that could also capture systematically the unanticipated ripple effects in the community or wider region, as those may be equally important in changing norms, capabilities and behavior of a community.

## Figures and Tables

**Table 1:** Domains of capacity building used for assessment.

Capacity Building Domain	Description/Definition
**Organizational structures**	Community members come together regularly in order to address their concerns and problems and establish links with others.
**Participation**	Community members are actively involved in stakeholder group meetings and activities.
**Leadership**	Participants of the stakeholder group take initiative and work with other groups to gain resources.
**Problem assessment and solution; skills**	The stakeholder group can identify problems and carries out actions to resolve the problems; the assessment is used to strengthen community planning.
**Critical awareness/reflexivity**	The stakeholder group can reflect on assumptions underlying their actions as well as self-analyze and improve their activities over time.
**Resource mobilization**	The stakeholder group can raise resources and decide on fair distribution.
**Links with others, networks**	The stakeholder group establishes partnerships and coalitions between their group and others, thereby generating resources and recruiting new members.
**Role of outside agents/power**	The facilitating health promotion researchers transform power relationships to the stakeholder group, such that the stakeholder group assumes authority and makes their own decisions.
**Program management**	The stakeholder group has clearly defined roles and responsibilities and can manage program development and implementation with little or no assistance of the facilitating researchers.

Legend: Domains of capacity building, as suggested by Laverack [44,47], and Sauter et al [40], also based on Gibbon et al. [45], Goodman et al. [49].

**Table 2:** Sample sizes for protocols and interviews retrieved from both communities.

	Total	Community A	Community B
Semi-standardized monitoring protocols			
Monitoring protocols	*N* = 23	*N* = 10	*N* = 13
Semi-standardized interviews with participants			
All interviews	*N* = 13	*N* = 8	*N* = 5
Interviews with participating stakeholders	*N* = 9	*N* = 6 (sport club representatives, municipal staff, city councilor)	*N* = 3 (sport club representatives)
Interviews with drop-outs	*N* = 4	*N* = 2 (representative of the adult education center, pharmacist)	*N* = 2 (director of a private gym, representative of workers’ comradeship association)

**Table 3:** Stakeholder group meetings and outcomes.

	Community A	Community B
**Stakeholder groups**		
**Number of meetings**	*N* = 10	*N* = 13
**Time period**	11 months	30 months (ongoing)
**Intervals**	Monthly	Monthly to quarterly
**Number of participants**	8–27 participants/meeting	7–30 participants/meeting
**Profession/role of long-term participants**	Sport club representatives, municipal staff, town councilor, local businesses	Sport club representatives, mayor, senior citizens’ representative
**Implemented interventions**		
**Number of interventions**	*N* = 2	*N* = 3
**Description of interventions**	1. “Website”: Advertising of courses and activities suitable for men 50+, across the local sport clubs, on the community homepage. 2. “Leaflets”: Design, print and distribution of flyers listing suitable courses for men 50+ across the local sport clubs.	1. “SportCard”: trial offer of a range of courses across sport clubs, at a small price without membership.
		2. “Outdoor Activity Meetings”: Free informal weekly meetings in community locations with varying exercise activities (walking, gymnastics etc.), run by a trainer
		3. “German Sports Insignia”: Training sessions for obtaining a badge for sporting achievements, awarded by the German Olympic Sports Federation, graded according to age and gender.

**Table 4:** Differences in capacity building between both communities.

	Community A	Community B
**Organizational structures**	Stakeholder groups could be implemented and met regularly They implemented several physical activity interventions	
	Meetings stopped after almost a year	Regular meetings were maintained over years A local ex-sports trainer was hired to manage the group
**Participation**	Diversity of group members decreased over time; mainly representatives of sport clubs and council remained Dominance of the sport clubs may have driven other participants out of the group members planned sport club-related interventions efficiently Some group members were doubtful about participatory approach	
**Leadership**	Group members were often passive; no considerable leadership was developed	The mayor took on a leadership role and set the agenda of meetings Later, an ex-sport trainer was employed to run the activities of the group; he developed leadership qualities
**Problem assessment and solution; skills**	Both groups were aware that older men have specific needs re. physical activity, the groups identified certain exercise barriers and succeeded in finding adequate solutions, but only with regard to sport clubs The stakeholders seemed reluctant to analyze other causes for older men’s inactivity	
**Critical awareness/reflexivity**	For the greatest part, groups appeared satisfied with the achievements.	
		The stakeholder group repeatedly discussed the acceptance of the SportCard and the novel sport courses
**Resource mobilization**	Both stakeholder groups mobilized necessary resources for the realization of the interventions (marketing, trainers, venues)	
		The municipality funded and subsidized many interventions, as the mayor was an active group member
**Links with others, networks**	Coalitions were mainly built with other sport clubs and the press	
**Role of outside agents/power; Program management**	The research team was consistently seen as responsible for most of the organizational tasks Stakeholder groups developed their own solutions after a few meetings, decisions were made jointly with the researchers.	
	After the first intervention (leaflet) was implemented, the group become more passive again	The stakeholders kept developing further strategies and decided independently on processes
